# Quality-on-Demand Compression of EEG Signals for Telemedicine Applications Using Neural Network Predictors

**DOI:** 10.1155/2011/860549

**Published:** 2011-07-03

**Authors:** N. Sriraam

**Affiliations:** Center for Biomedical Informatics and Signal Processing, Department of Biomedical Engineering, SSN College of Engineering, Chennai 603110, India

## Abstract

A telemedicine system using communication and information technology to deliver medical signals such as ECG, EEG for long distance medical services has become reality. In either the urgent treatment or ordinary healthcare, it is necessary to compress these signals for the efficient use of bandwidth. This paper discusses a quality on demand compression of EEG signals using neural network predictors for telemedicine applications. The objective is to obtain a greater compression gains at a low bit rate while preserving the clinical information content. A two-stage compression scheme with a predictor and an entropy encoder is used. The residue signals obtained after prediction is first thresholded using various levels of thresholds and are further quantized and then encoded using an arithmetic encoder. Three neural network models, single-layer and multi-layer perceptrons and Elman network are used and the results are compared with linear predictors such as FIR filters and AR modeling. The fidelity of the reconstructed EEG signal is assessed quantitatively using parameters such as PRD, SNR, cross correlation and power spectral density. It is found from the results that the quality of the reconstructed signal is preserved at a low PRD thereby yielding better compression results compared to results obtained using lossless scheme.

## 1. Introduction

Electroencephalography (EEG) is widely used in brain research and clinical diagnosis [1–3]. Due to the enormous data size of the EEG resulting from large electrode arrays and prolonged recordings, data compression is desired for efficient data archiving and transmission through networks. Furthermore, for telemedicine or telebrowsing applications, transmitting a large amount of digital data through a bandwidth-limited channel becomes a heavy burden. Compression techniques practically aim at obtaining maximum data volume reduction while preserving the significant feature upon reconstruction. Data compression can be lossless, when the signal waveform fidelity is totally preserved, or lossy, in cases where a certain amount of distortion or lack of fidelity in the decompressed data is allowed. Efficient compression of the EEG signal is a difficult task due to the randomness inherent in the signal, and hence high compression rates cannot be achieved with lossless compression methods [4–10]. In [10], an adaptive error modeling technique for lossless compression has been applied to improve the compression efficiency. In [11], Wongsawat et al. have shown the application of KLT transform for the lossless compression of EEGs. A context-based error model using linear and neural network predictors has shown the removal of offset bias for attaining some improvement in compression efficiency [6, 12]. The effect of uniform quantization and nonuniform quantization on compression gain using the near-lossless compression of EEG signals has been reported in [6, 13, 14]. Gopikrishna and Makur proposed a near-lossless compression scheme using wavelets and ARX model [15]. In [16], The author has shown the influence of context-based error modeling for the near-lossless compression of EEG signals.

Lossy compression techniques might be acceptable as long as the reconstructed quality of the signal preserves the diagnostic information for clinical investigation [17–19]. Further, recent works reported based on pursuit approach with wavelet dictionaries, wavelet-SPIHT, and finite rate of innovation technique exploiting sampling theory have shown some improvement in the compression performance [20–22]. It can be revealed from the literature that the compression schemes for ECG signals have emphasized the quality aspects of the reconstructed signal at lower bandwidth utilization [23–26]. To the best of the authors knowledge, attempts have not been made to interpret the quality aspects of the EEG signal for telemedicine applications and at the same time satisfying the low-bandwidth requirement. This paper discusses the quality on demand compression of EEG Signal using neural network predictors. Three neural network models, namely, single-layer perceptron (SLP), multi-layer perceptron (MLP), and Elman network (EN), are used [7–10], and the performances are evaluated in terms of bits per sample (BPS) and fidelity parameters such as percent of root mean square difference (PRD), signal-to-noise ratio (SNR), cross-correlation (CC), and power spectral density (PSD). The results are also compared with two linear predictors, namely, finite impulse response (FIR) filters and autoregressive (AR) model.

## 2. Quality on Demand Compression

**Figure 1:**
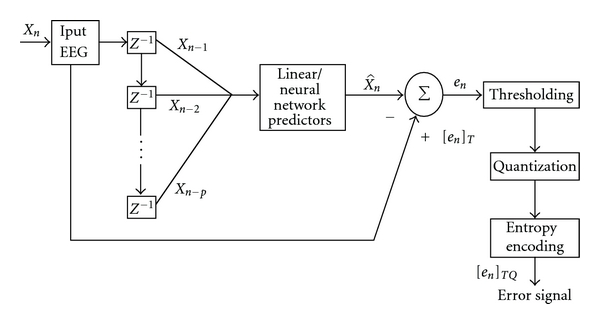
Quality on demand compression scheme.

It is well known that a higher compression can be achieved by sacrificing the quality of the reconstructed signal and vice versa. A trade-off has to be made to obtain a good quality of decoded signal with a considerable amount of compression. For telemedicine applications, a physician at the receiving end must interactively adjust certain parameters associated with compression algorithm according to physician's quality consideration. It has been reported in [12, 23–26] that two factors, namely, bits per sample (BPS) and percent of root-mean-square-difference (PRD) decide the quality on demand specifications, namely, bandwidth constraints and reconstructed signal quality, respectively. This paper highlights the quality on demand compression scheme for EEG signal using neural network predictors. The fidelity of the reconstructed signal is measured quantitatively by means of four factors, namely, PRD, SNR, CC, and PSD. For EEG signal compression, two-stage lossless compression schemes involving predictor in the first stage with an entropy encoder in the second stage have been successfully used [6–10]. The main function of the predictor is to estimate the present value of a sample using its past samples and then transmit only the error (residues), which are generally of a lesser magnitude and size than the original samples. It is assumed that both the encoder and the decoder simulate an identical prediction process [27]. The prediction process starts with the transmission of initial header information consisting of neural network parameter settings and selected number of input sample values. At the receiving end, the prediction process is repeated and the original input is recovered by adding the transmitted residues to the predicted values. If we transmit the error signals based on certain threshold values and followed by quantization, there is a possibility of achieving better compression, and it may also be clinically acceptable as long as the reconstructed signal preserves the required diagnostic features. The compression efficiency can be further improved by using an arithmetic entropy encoder in the second stage [28]. For a quality on demand compression of EEG signal, the two-stage compression scheme as reported in [7–10, 13] can be modified as shown in Figure 1.

If *X*_*n*_ is the current sample and ${\hat{X}}_{n}$ is the predicted sample, then the value of the error (residue) sample is given by  (1)

(1) $$e_{n}=X_{n}-{\hat{X}}_{n}.$$

Figure 1 shows the block diagram of the proposed compression scheme. The thresholded value of *e*_*k*_ denoted as [*e*_*n*_]_*T*_ is determined based on a threshold value *T* as shown in (2).

(2) $${\left[e_{n}\right]}_{T}=\{\begin{array}{cc} 0, & \text{if}\;\left|e_{n}\right|<T\\ e_{n}, & \text{if}\;\left|e_{n}\right|\geq T \end{array}$$

According to (2), if the magnitude of the error signal *e*_*n*_ is less than the threshold value *T*, it is assumed to be zero. On the other hand, if the magnitude of the error signal is greater than or equal to *T*, the actual value of error signal is transmitted. The error signals are then quantized and encoded using an arithmetic encoder which is denoted as [*e*_*n*_]_*TQ*_. At the receiving end, the output ${\hat{X}}_{n}$ of the counterpart neural network combined with the transmitted error signal [*e*_*n*_]_*TQ*_ to obtain the resultant signal ${\tilde{X}}_{n}$ is shown below

(3) $${\tilde{X}}_{n}={\hat{X}}_{n}+{\left[e_{n}\right]}_{TQ},$$

where ${\tilde{X}}_{n}$, is the resultant signal and [*e*_*n*_]_*TQ*_, is the thresholded and quantized value of the error signal. 

The performance of two-stage compression scheme is evaluated for different values of *T*.

## 3. Neural Network Predictors

**Figure 2:**
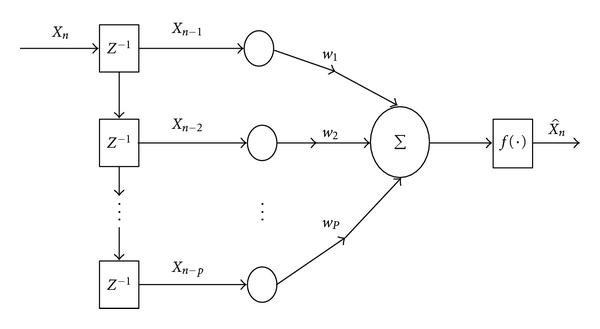
A *P*-th order single-layer perceptron network.

**Figure 3:**
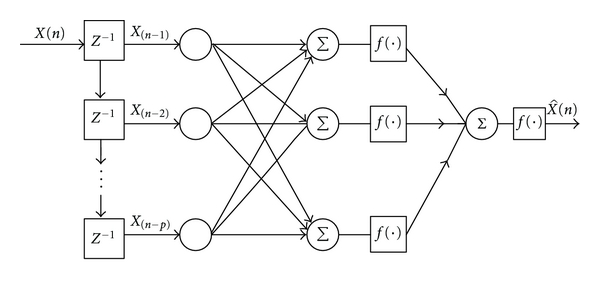
A *P*-th order multi-layer perceptron network.

**Figure 4:**
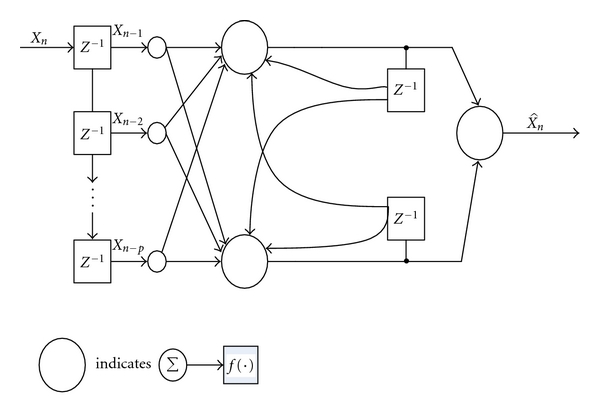
A *P*-th Elman network model.

Neural networks possess certain attractive properties such as massive parallelism, robustness, adaptive learning, self-organization, fault tolerance, and generalization which are useful to enhance the performance of a predictor [29]. The purpose of the predictor is to decorrelate the input data thereby reducing the amplitude range of the data and generating a sequence, which is approximately white Gaussian. In this paper, the neural network models considered are: (1) single-layer perceptron (SLP), (2) multi-layer perceptron (MLP), (3) Elman network (EN). The architectures of SLP, MLP, and EN with *P*-th predictor order are shown in Figures 2, 3, and 4, respectively [27, 30]. The first two networks are feed forward models whereas the third one is a feedback network. 

All the neural network predictors are optimally configured based on the parameters, such as number of hidden neurons, predictor order, activation functions, learning algorithms in order to ensure the network convergence with minimum error.  Table 1 shows the configuration details along with the number of iterations required during the training phase [10, 16].

## 4. Experimental Setup

**Figure 5:**
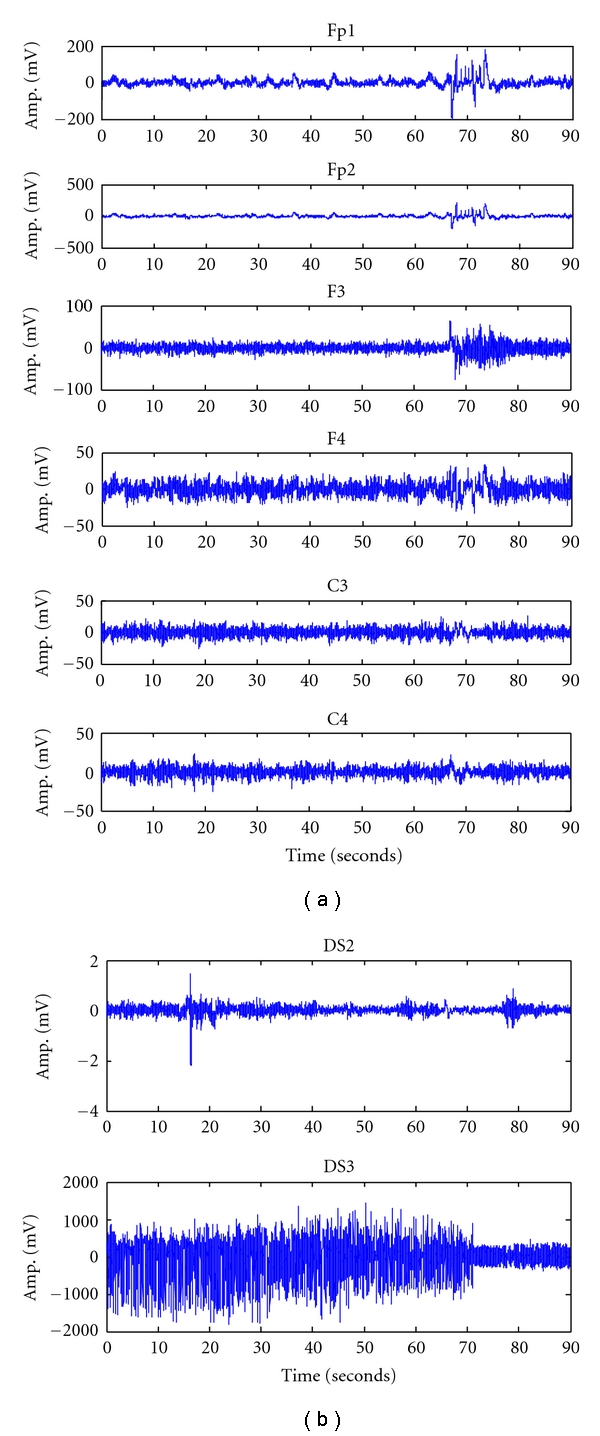
Recordings of different EEG signals: (a) epileptic with sudden seizure and (b) normal and pure epileptic signal.

For our experimental study, we have used EEG signals recorded under three different physiological conditions, namely, EEG dataset1 (DS1) which consists of a 16-bit EEG signal recorded under epileptic condition with sudden seizures obtained from six channels (Fp1, Fp2, F3, F4, C3, C4) with a sampling rate of 256 Hz, EEG dataset2 (DS2) which consists of a 16 bit normal EEG signal with eyes open and closed, recorded using BIOPAC data acquisition system with a sampling rate of 256 Hz [10], and EEG dataset3 (DS3) which contains EEG signals recorded during the occurrence of epileptic seizures exhibiting ictal activity with a sampling rate of 173.61 Hz [10, 31]. Figure 5 shows exemplary EEG signals corresponding to the three datasets described above.

## 5. Performance Evaluation Parameters

The performances of the proposed compression schemes are evaluated using the compression parameter, compression ratio (CR), and four fidelity parameters, namely, percent of root-mean-square-difference (PRD), signal-to-noise ratio (SNR), peak signal signal-to-noise ratio (PSNR), cross correlation (CC), and power spectral density (PSD). 

The compression ratio (CR) is defined as follows [27]:

(4) $$\text{C}{\text{R}}_{}=\frac{vn}{pn+wx+\left(v-p-q\right)r},$$

where

*v*: total number of samples in test file,
*n*: total number of bits used to represent a sample,
*p*: order of the predictor,
*q*: number of residues below *T*,
*w*: number of weights,
*x*: number of bits used to represent a weight,
*r*: number of bits used to represent a residue.

To validate the reliability of the compression method, the fidelity (quality) of the reconstructed signal has to be assessed. The parameters, PRD, SNR, PSNR, CC, and PSD are used to judge the quality of the reconstructed EEG signal.

PRD is defined as [32, 33]

(5) $$\text{PRD}={\sqrt{\frac{\sum_{n=1}^{N}{\left(X_{n}-{\tilde{X}}_{n}\right)}^{2}}{\sum_{n=1}^{N}{\left(X_{n}\right)}^{2}}}}^{}\cdot 100,$$

where

*X*_*n*_: is the original sample,
${\tilde{X}}_{n}$: is the reconstructed sample,
*N*: is the length of the window over which the PRD is calculated.

SNR is defined as shown [33]:

(6) SNR=−20log   (0.01 PRD)

PSNR is defined as shown [13]:

(7) $$\text{PSNR}=20\log\;\left[\frac{\max\;\left(X_{k}\right)}{\text{RMSE}}\right],$$

where max (*X*_*k*_) is the maximum value of the original EEG signal

RMSE is the root mean square error which is defined as shown

(8) $$\text{RMSE}=\sqrt{\frac{1}{N}{\left(\text{PRD}\right)}^{2}\sum {\left(X_{k}\right)}^{2}}.$$

Cross correlation (CC) denotes the statistical correlation between two signals [34]. The correlation between the original and the reconstructed signal is measured by CC which is defined as

(9) $$\text{CC}=\frac{\mathrm{cov}\;\left(X_{n}-{\tilde{X}}_{n}\right)}{\sqrt{\text{var}\left(X_{n}\right)\cdot \text{var}\left({\tilde{X}}_{n}^{}\right)}},$$

where $\mathrm{cov}\;(X_{n}-{\tilde{X}}_{n})$ is the covariance between *X*_*n*_ and ${\tilde{X}}_{n}$var(*X*_*n*_) and $\text{var}({\tilde{X}}_{n})$ are the variances of *X*_*n*_ and ${\tilde{X}}_{n}$, respectively. 

Cross correlation plays an important role in judging the resemblance of two signals. It can be concluded that the reconstructed signal is very close to the original one when cc = 1.

Power spectra of original and reconstructed EEG signals is calculated by determining the parameter PSD [35, 36]. The similarities in both the power spectra imply that the original and reconstructed EEG signals are approximately identical. 

The error signal *e*_*n*_ obtained from the difference between the original signal and predicted EEG signals are thresholded by varying *T* in accordance to the 1–10% of the maximum value of *e*_*n*_. The thresholded error signals are further quantized in to three levels, namely, Q1, Q2, and Q3. For the experimental data sets, the bits assigned for the three quantization levels (QL) are shown in Table 2.

## 6. Experimental Results

The performance of the proposed compression scheme is evaluated in terms of CR by varying the threshold, *T*, and quantization levels, QL, using the two-stage compression with different predictors in the first stage and an arithmetic encoder in the second stage. Tables 3, 4, and 5 show the values of CR obtained for the three experimental datasets DS1, DS2 and DS3 at Q1, Q2, and Q3, respectively. For DS1, the mean values of CR obtained from all six channels are given.

From Tables 3, 4, and 5, it is found that the CR value increases as *T* increases. Further, the effect of compression increases as the QL increases. It can be seen that the values of CR obtained at Q3 yield the best compression results. Among the predictors, SLP yields the best results for all the three datasets used for this experimental study.

**Figure 6:**
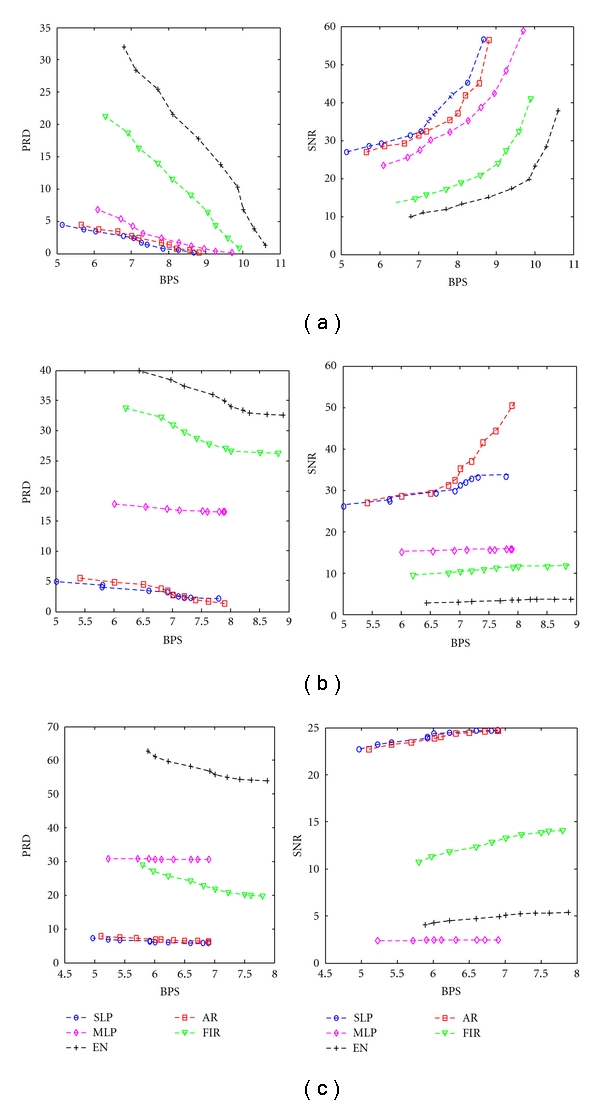
BPS versus PRD and BPS versus SNR characteristics at different quantization levels, (a) Q1, (b) Q2, and (c) Q3.

 The quality of the reconstructed signal at different thresholds is evaluated in terms of PRD and SNR for the two-stage compression schemes.  Figure 6 shows the variation of PRD and SNR with respect to BPS at different *T* and quantization levels using different predictors for the DS1. The PRD and SNR values represent the mean values obtained for the reconstructed EEG signals of all the six channels. 

It is found from Figure 6 that the increase in BPS results in the decrease of PRD (increase of SNR) value. Among the different prediction scheme, SLP yields the best results. It can be seen that the value of BPS decreases as QL increases. It can be concluded that the minimum BPS obtained at Q1, Q2 and Q3 are 5.32, 5.02, and 4.9, respectively.

**Figure 7:**
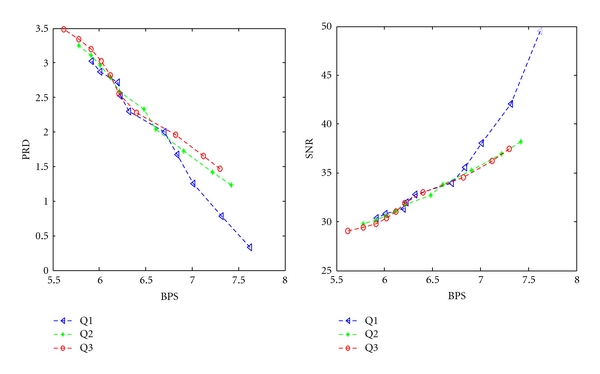
BPS versus PRD and BPS versus SNR characteristics using DS2.

**Figure 8:**
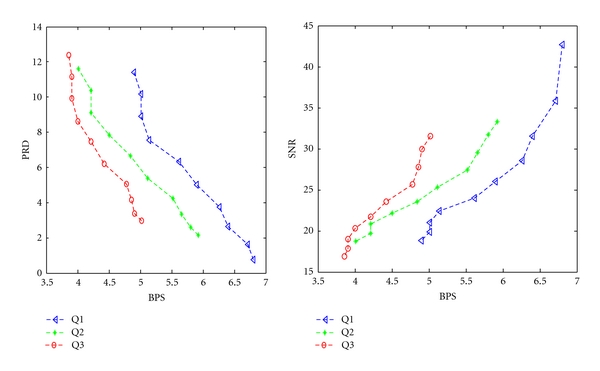
BPS versus PRD and BPS versus SNR characteristics using DS3.

Figures 7 and 8 show the variations of PRD and SNR with BPS for DS2 and DS3, respectively, using the SLP.

**Figure 9:**
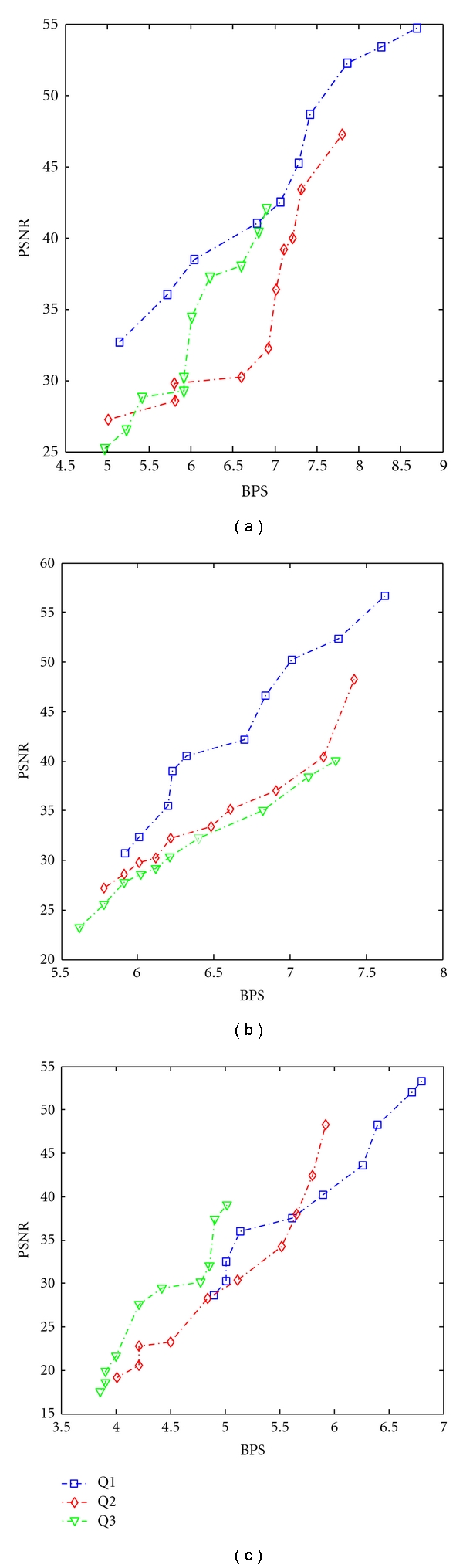
Plot of BPS versus PSNR for (a) DS1, (b) DS2, and (c) DS3.

From Figure 7, It is found that the minimum BPS obtained at Q1, Q2, and Q3 are 5.92, 5.72, and 5.3, respectively. From Figure 8, It is found that the minimum BPS obtained at Q1, Q2, and Q3 are 5.02, 4.3, and 4.01, respectively.  Figure 9 shows the fidelity plot in terms of PSNR using SLP predictor.

The quality of the reconstructed signal is then further assessed by estimating the parameters, CC and PSD. The value of CC is evaluated for the three EEG datasets at different QL.  Table 6 shows the CC obtained using SLP which yields the best results.

From Table 6, it is found that the CC value decreases as the value of threshold, *T*, increases. The correlation between the original and the reconstructed signal increases as the quantization increases. This holds good for all the three experimental data sets.

**Figure 10:**
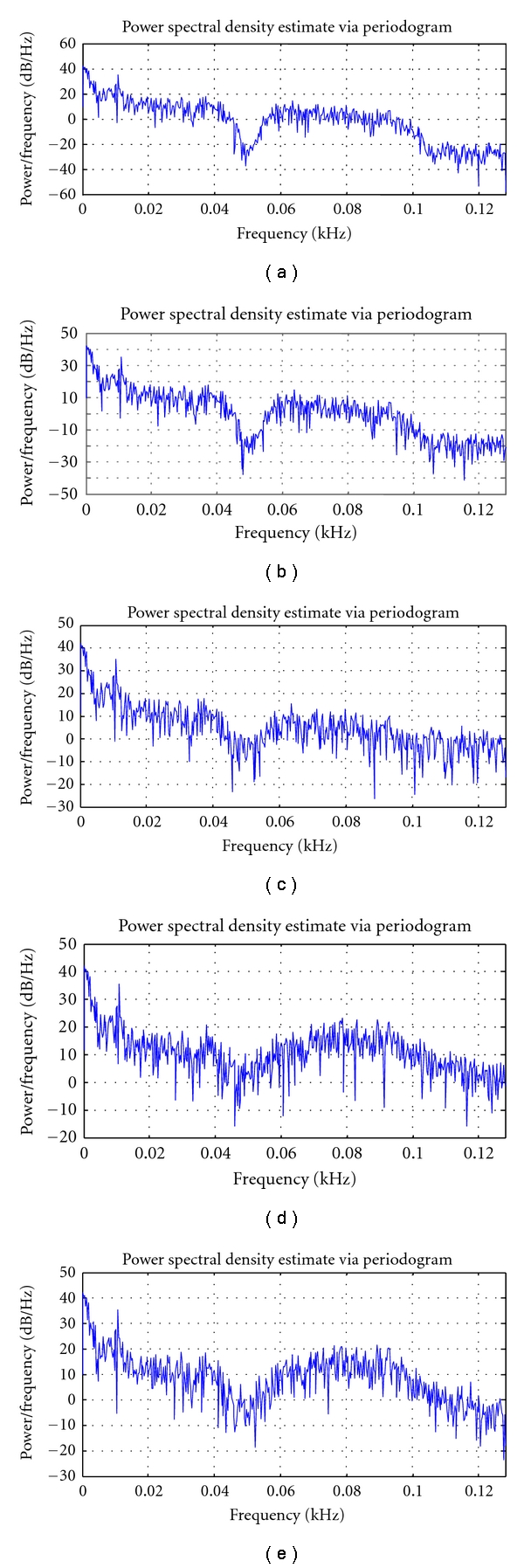
Power spectra of EEG dataset1: (i) original signal, (ii) reconstructed signal at *T* = 4 at Q1, (iii) reconstructed signal at *T* = 7 at Q1, (iv) reconstructed signal at *T* = 4 at Q2, and (v) reconstructed signal at *T* = 3 at Q3.

**Figure 11:**
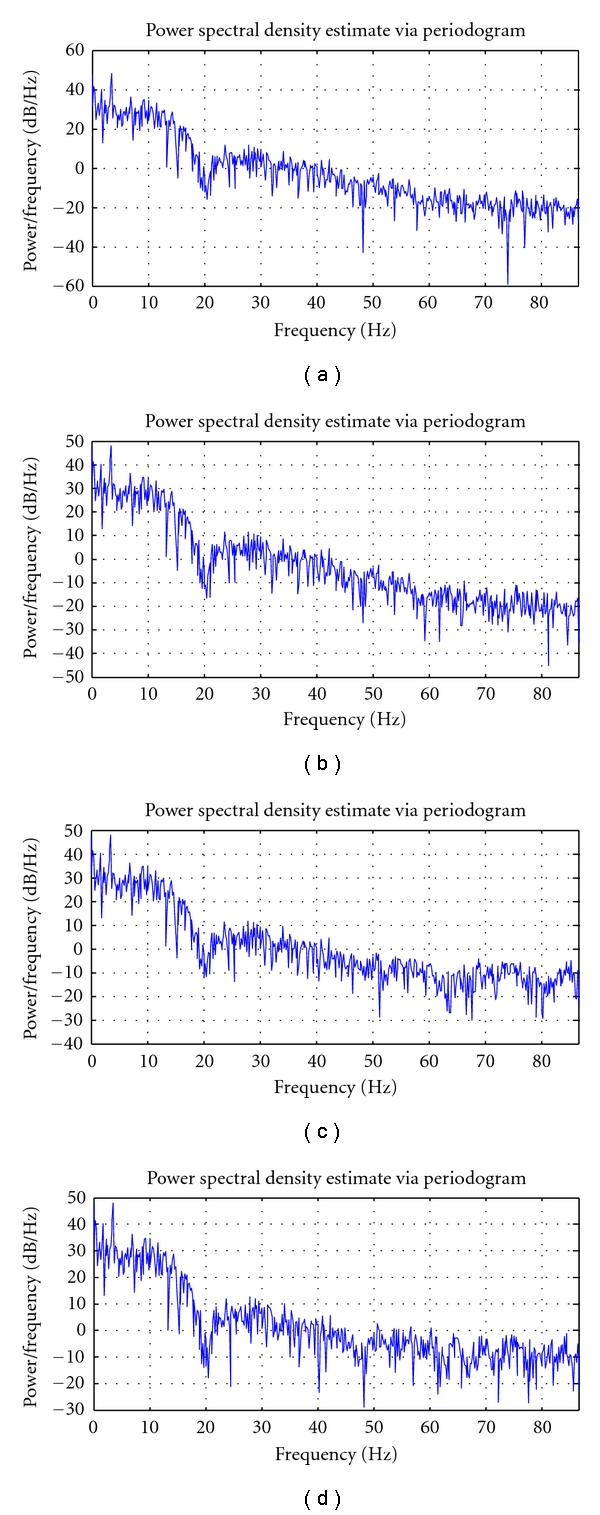
Power spectra of EEG dataset2: (i) original signal, (ii) reconstructed signal at *T* = 7 at Q1, (iii) reconstructed signal at *T* = 5 at Q2, and (iv) reconstructed signal at *T* = 5 at Q3.

**Figure 12:**
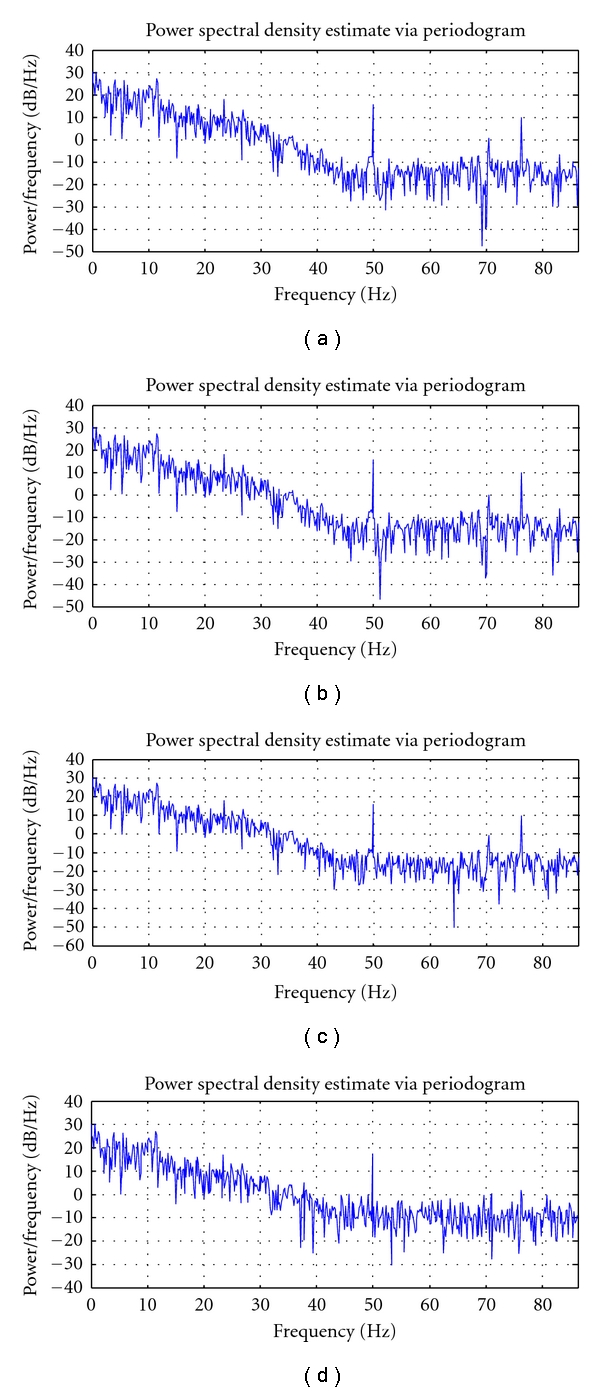
Power spectra of EEG dataset3: (i) Original signal, (ii) reconstructed signal at *T* = 5 at Q1, (iii) reconstructed signal at *T* = 3 at Q2, and (iv) reconstructed signal at *T* = 3 at Q3.

Figures 10–12 depict the PSD estimation of the original signal and the reconstructed signal for the three datasets.

From Figure 10, it is found that for the dataset DS1, the PSD of the reconstructed signal until *T* = 6, *T* = 4, and *T* = 3 resembles the original signal for the quantization levels Q1, Q2, and Q3, respectively. From Figure 11, it is found that for the dataset DS2, the PSD of the reconstructed signal until *T* = 7, *T* = 5, and *T* = 3 resembles the original signal for the quantization levels Q1, Q2, and Q3, respectively. From Figure 12, it is found that for the dataset DS3, the PSD of the reconstructed signal until *T* = 6, *T* = 4, and *T* = 2 resembles the original signal for the quantization levels Q1, Q2, and Q3, respectively.

## 7. Discussion

The main idea of the proposed compression scheme is to obtain the reconstructed EEG signal suitable for clinical diagnosis at a lower BPS and PRD. The effect of thresholding and quantization level decides the quality on demand criteria, and a better trade-off between the BPS and PRD is achieved for the clinical investigations. Suppose the enduser wishes to receive the quality of the reconstructed signal at certain PRD, then the parameters BPS, *T*, QL, and CC for the three experimental datasets can be chosen accordingly as shown in Table 7.

From Table 7, it can be observed that an average compression efficiency [13] of 74.12%, 76.2%, and 79.2% is achieved, respectively, for the three specified fidelity criterions. For telemedicine-based transmission and retrieval, one can ensure the diagnostic quality of the reconstructed EEGs based on appropriate selection of the fidelity criteria with efficient low-bandwidth utilization. It can be further seen from the results shown in Table 7 that the proposed scheme was found to yield comparable results with the near-lossless scheme reported in [13].

## 8. Conclusions

This paper discusses the quality on demand compression scheme for EEG signal using neural network predictors. Neural network predictors such as single-layer perceptron, multi-layer perceptron, and Elman networks have been used. The error signals were thresholded before they were applied to the arithmetic encoder. A two-stage compression scheme with a predictor in the first stage and an entropy encoder in the second stage has been used. The error (residue) signal which is the difference between the original and the predicted EEG signals was first thresholded using various levels of thresholds and was further quantized and then encoded using an arithmetic encoder. Three neural network models, namely, single-layer perceptron, multi-layer perceptron, and Elman network were used, and the results were compared with linear predictors such as FIR filters and AR modeling. Experiments were carried out using EEG signals recorded at various physiological conditions. The fidelity of the reconstructed EEG signal was assessed quantitatively using parameters such as percent of root- mean-square- difference (PRD), signal-to-noise ratio (SNR), cross correlation (CR) and power spectral density (PSD). It has been found from the experimental results that the single-layer perceptron yields the best results by preserving the diagnostic information at low PRD values.

## Figures and Tables

**Table 1:** Optimal configuration of the network.

Type of neural network	Predictor order	No. of hidden neurons	Activation function		Learning algorithm	No. of epochs required for training (only average is shown)
			Hidden layer	Output layer		
SLP	5	—	—	Linear	Levenberg-Marquart (LM)	65
MLP	3	5	Log-sigmoid	Linear	Levenberg-Marquart (LM)	230
EN	5	5	Tan-sigmoid	Linear	Gradient descent with momentum and adaptive learning rate back propogation (GDX)	750

**Table 2:** Quantization level.

EEG datasets	Q1	Q2	Q3
DS1	9	8	7
DS2	8	7	6
DS3	6	5	4

**Table 3:** Values of CR obtained at Q1.

EEG data set	*T* (%)	SLP	MLP	EN	AR	FIR
DS1	1	3.87	3.58	3.46	3.78	3.55
	3	3.98	3.58	3.52	3.84	3.58
	5	4.06	3.78	3.52	3.87	3.63
	7	4.09	3.78	3.63	3.98	3.67
	9	4.26	3.84	3.68	3.98	3.67

DS2	1	3.81	3.57	3.38	3.79	3.41
	3	3.89	3.59	3.38	3.81	3.49
	5	4.01	3.59	3.41	3.81	3.49
	7	4.14	3.6	3.47	3.92	3.5
	9	4.14	3.61	3.47	3.92	3.5

DS3	1	3.64	3.49	3.27	3.61	3.29
	3	3.77	3.52	3.38	3.64	3.45
	5	3.97	3.59	3.49	3.82	3.54
	7	4.33	3.8	3.66	3.95	3.71
	9	4.42	3.89	3.73	4.18	3.79

**Table 4:** Values of CR obtained at Q2.

EEG data set	*T* (%)	SLP	MLP	EN	AR	FIR
DS1	1	3.95	3.75	3.58	3.87	3.68
	3	4.22	3.87	3.67	3.98	3.76
	5	4.39	3.98	3.72	4.07	3.82
	7	4.67	4.14	3.72	4.21	3.87
	9	4.87	4.14	3.84	4.48	3.96

DS2	1	3.81	3.57	3.38	3.79	3.41
	3	3.89	3.59	3.38	3.81	3.49
	5	4.01	3.59	3.41	3.81	3.49
	7	4.14	3.6	3.47	3.92	3.5
	9	4.14	3.61	3.47	3.92	3.5

DS3	1	3.64	3.49	3.27	3.61	3.29
	3	3.77	3.52	3.38	3.64	3.45
	5	3.97	3.59	3.49	3.82	3.54
	7	4.33	3.8	3.66	3.95	3.71
	9	4.42	3.89	3.73	4.18	3.79

**Table 5:** Values of CR obtained at Q3.

EEG data set	*T* (%)	SLP	MLP	EN	AR	FIR
DS1	1	4.22	3.84	3.78	3.98	3.76
	3	4.44	3.96	3.87	4.16	3.84
	5	4.81	4.08	3.98	4.27	3.93
	7	4.89	4.22	4.04	4.42	4.04
	9	5.06	4.21	4.1	4.54	4.16

DS2	1	4.09	3.7	3.53	3.97	3.71
	3	4.25	3.7	3.57	4.0	3.79
	5	4.37	3.92	3.65	4.09	3.79
	7	4.39	3.95	3.65	4.2	3.81
	9	4.49	4.01	3.65	4.31	3.81

DS3	1	4.39	4.21	3.78	4.34	3.78
	3	4.44	4.24	3.98	4.37	4.07
	5	4.71	4.41	4.13	4.56	4.24
	7	5.09	4.5	4.2	4.86	4.35
	9	5.21	4.7	4.34	5.05	4.44

**Table 6:** Estimation of CC using different two-stage schemes.

EEG data set	*T* (%)	Q1	Q2	Q3
DS1	1	0.982	0.958	0.923
	3	0.972	0.93	0.875
	5	0.964	0.921	0.842
	7	0.942	0.90	0.83
	9	0.891	0.865	0.81

DS2	1	0.99	0.98	0.976
	3	0.987	0.97	0.962
	5	0.965	0.952	0.92
	7	0.95	0.93	0.90
	9	0.921	0.91	0.89

DS3	1	0.984	0.97	0.965
	3	0.974	0.961	0.951
	5	0.932	0.91	0.88
	7	0.92	0.89	0.862
	9	0.90	0.887	0.86

**Table 7:** Selection of quality criteria parameters.

EEG data sets	PRD <1.0				1≤ PRD< 3				3≤ PRD< 5			
	PSNR≥ 52 db				40 db≤PSNR≤50 db				PSNR≤ 40db			
	BPS	*T*	QL	CC	BPS	*T*	QLLL	CC	BPS	*T*	QL	CC
DS1	7.95	2	Q1	0.98	6.52	6	Q2	0.92	5.62	7	Q3	0.83
DS2	7.01	3	Q1	0.965	5.92	8	Q2	0.92	5.71	6	Q3	0.89
DS3	6.62	2	Q1	0.98	5.5	3	Q2	0.96	4.32	5	Q3	0.88
